# Re-assessing the total burden of norovirus circulating in the United Kingdom population

**DOI:** 10.1016/j.vaccine.2017.01.009

**Published:** 2017-02-07

**Authors:** John P. Harris, Miren Iturriza-Gomara, Sarah J. O'Brien

**Affiliations:** National Institute for Health Research Health Protection Research Unit (NIHR HPRU) in Gastrointestinal Infections at the University of Liverpool, Liverpool, UK

**Keywords:** Norovirus, Gastrointestinal infections, Calicivirus, Infectious diseases

## Abstract

The second Infectious Intestinal Diseases study (IID2) estimated the incidence of norovirus in the UK at 47/1000 population (three million cases annually). Clinically significant norovirus was defined using a cycle threshold (ct) value of <30; a more stringent cut-off than used in diagnostic laboratories. The low infectious dose of norovirus means asymptomatic individuals potentially contribute to ongoing transmission. Using a less stringent but diagnostically relevant threshold increases the estimation of the population burden of norovirus infection by around 26% to 59/1000 person years (95% CI 52.32–64.98), equating to 3.7 million norovirus infections annually (3.3–4.1 million). With possible vaccines on the horizon for norovirus, having a good estimate of the total burden of norovirus infection, as well as symptomatic disease will be useful in helping to guide vaccination policy when candidate vaccines become available.

## Highlights

• Norovirus is the commonest cause of GI disease in the UK.
• Less stringent diagnostic threshold Increases previous estimate by 26%.
• Re-assessed estimate equates to burden of infection at 59 per 1000 person years.

## 1 Introduction

Norovirus is the commonest cause of gastrointestinal disease across all age groups worldwide [1]. The majority of cases experience a mild, self-limiting illness and few cases tend to consult primary healthcare. Those that do might not be sampled, leading to huge under-diagnosis and under-reporting [2]. The Second Study of Infectious Intestinal Disease in the community (IID2 study) in the UK estimated the community incidence of norovirus to be 47/1000 population, which equates to around three million cases a year [3], at a cost to cases and the health service of up to £106million (around US $178 million at that time) [4].

The IID2 study is one of only two large population based cohort studies in Europe that provided disease estimates using molecular methods for detecting norovirus in stool samples [3], [5]. In the IID2 study clinically significant norovirus was defined by using a cycle threshold (ct) value of 30 or less [3]. This was a more stringent cut-off than would normally be employed in clinical diagnostic laboratories. Norovirus detected in stool samples above a ct value of 30 was considered to reflect asymptomatic carriage or sub-clinical infections [6]. Thus the estimates of norovirus using a ct value of 30 or less from the IID2 study equated to the burden of symptomatic disease.

The role of asymptomatic carriage of norovirus, particularly in causing outbreaks or ongoing transmission events, is unclear [7]. The very low infectious dose of norovirus makes it a distinct possibility that asymptomatic individuals contribute to ongoing transmission but the evidence is mixed. In one large hospital outbreak there was no apparent correlation between ct value and symptom duration or onward transmission [8] and a study of outbreaks in healthcare settings found asymptomatic healthcare workers were rarely involved in transmission [9]. However there are documented outbreaks that support this hypothesis [10], [11]. Given the potential public health significance of asymptomatic carriage we have re-assessed the total burden of circulating norovirus in the UK population by using the higher ct value of <40 for norovirus.

## 2 The study

The IID2 study has been described in detail elsewhere [3], [12], [13]. Briefly, one of the components of the study comprised a prospective cohort study where randomly selected healthy people of all ages were enrolled from randomly selected general practices (GP) across the United Kingdom. Volunteers were followed up at weekly intervals for one year to identify any symptoms of infectious intestinal disease (IID).

People who fulfilled the case definition i.e. people who developed clinically significant vomiting (more than once in a 24 h period, or it caused incapacity or was accompanied by other symptoms) or loose stools for a period of less than two weeks, without a known non-infectious cause and who had previously been symptom free in the preceding three weeks, were asked to complete a clinical symptom questionnaire. The definition of vomiting excluded conditions associated with non-infectious causes such as pyloric stenosis or morning sickness. All cases were asked to provide a stool sample for microbiological testing. Diagnosis of clinically relevant norovirus used real time quantitative reverse transcription polymerase chain reaction (RT-PCR) with a cycle threshold of less than 30. Here, however, we use the assay analytical cut off of less than 40 to estimate the likely total burden of circulating norovirus.

For norovirus-associated infection the incidence was derived by dividing the number of cases with a PCR-confirmed diagnosis, by the number of person years at risk in the cohort study. For specific age groups this is the number of cases in each age group divided by the number of person years at risk in each age group.

Not all participants submitted a stool specimen for diagnosis. To establish the status of the individual for norovirus-associated infection where a sample was missing (35% of cases), it was derived by imputation. The imputation method used Multiple Imputation by Chained Equations and modelled whether the individual had a norovirus-associated infection as a function of age, sex and whether they exhibited any of five symptoms, vomiting, diarrhoea, bloody diarrhoea or fever. The imputation process created 20 datasets and burden of infection was estimated as the average number of infected individuals from the 20 imputed datasets. The number of people affected varied between the imputed datasets. In order to account for this variation, the imputation method was conducted 1000 times and the average values of the 20 imputed data sets were calculated on each iteration. The 5th and 95th centile of these averages were used for the credibility interval. The overall incidence rate and those for the age groups; under 1 year, 1–4 years, 5–14 years 15–64 years and 65 years and above were calculated. The person years at risk from the IID2 study were used as denominators.

Statistical analysis was conducted using R statistical software [14] using the MICE package [15].

A favourable ethical opinion to perform the IID2 Study was granted by the NHS North West Research Ethics Committee (07/MRE08/5) and all participants gave informed written consent prior to being enrolled in the study. No ethical review was required for this secondary analysis of fully anonymised data.

The overall burden of infection for norovirus-associated infections from the cohort study was 59 per 1000 person years (95% CI 52.32–64.98). Using the Office for National Statistics (ONS) mid 2009 population estimate for the UK, this equates to 3.7 million people infected with norovirus in a year (3.3–4.1 million).

The highest rates were in the youngest age groups (Table 1). The incidence in those aged less than 1 year was 238 per 1000 person years (186.11–446.65) and those aged 1–4 years the rate was 180 per 1000 person years (141.53–241.12). For children aged less than 5 years the rate was four times higher than all other age groups. The results of this study suggest that around 6% of the population and around 18% of children aged less than five years are affected by norovirus each year. The number of infections estimated was low particularly in the youngest age groups and this is reflected in the wide ranges in the estimated values.

## 3 Conclusions

The IID2 study provided estimates of clinically relevant norovirus disease, and suggested that around 3 million cases occur each year. This re-analysis, using a less stringent ct value of 40, adds around 800,000 cases of infection bringing the annual total of circulating norovirus closer to 4 million cases in the UK. This difference is important, especially in light of the candidate norovirus vaccines that are being developed. It is not yet clear how a vaccine might be implemented (if at all) and so to have good estimates of the total burden of norovirus infection, as well as symptomatic disease [16], should be useful in helping to guide vaccination policy when the time comes [17].

Key strengths of the IID2 study were its prospective nature, with active of follow-up and sampling of people declaring symptoms of IID, and the use of RT-PCR for confirming the presence of norovirus. Important limitations were a relatively low overall participation rate, although people who enrolled in the study tended to comply with the study procedures (including providing samples), and the lack of a formal control group. A limitation of the RT-PCR method is that it cannot distinguish between infectious and non-infectious virus.

In conclusion we have found that using a less stringent but diagnostically relevant threshold increases the estimation of the population burden of norovirus infection by around 26%. The additional burden in children aged under 5 was almost 30%. Given the possibility of a vaccine on the horizon [17], and the need for more robust estimates of the number of infections for policy makers, this study shows that the additional burden is greater in young children.

## Funding

This work was supported by the National Institute for Health Research Health Protection Research Unit in Gastrointestinal Infections at the University of Liverpool. The research was funded by the National Institute for Health Research Health Protection Research Unit (NIHR HPRU) in Gastrointestinal Infections at the University of Liverpool in partnership with Public Health England (PHE); University of East Anglia; University of Oxford and the Institute of Food Research [Grant number NIHR HPRU 2012-10038].

The IID2 Study was originally funded by the United Kingdom Food Standards Agency and the Department of Health [Grant number FS231043 (B18021)].

## Disclaimer

The views expressed are those of the authors and not necessarily those of the National Health Service, the National Institute for Health Research, the Department of Health or Public Health England.

## Conflict of interest

All authors declare no conflict of interest.

## Figures and Tables

**Table 1:** Age-specific burden of total circulating norovirus in the United Kingdom population, 2009.

Age group	No. infected	Person years	Infections/1000 person years (95%CI)	Infections/1000 person years from IID2 study^a^
All ages	276	4658.6	59.64 (52.3–64.9)	47 (39.1–56.5)
<1	6	26.9	238.02 (186.1–446.6)	178.2 (70.5–450.0)
1–4	34	190.8	179.83 (141.5–241.1)	137.3 (92.6–203.4)
5–14	28	424.1	66.65 (40.1–101.4)	59.6 (36.8–96.5)
15–64	126	2647.8	47.55 (34.7–58.2)	39.0 (31.3–48.7)
65+	45	1369.1	32.66 (20.4–45.3)	27.7 (19.6–39.1)
⩾5	40	217.6	183.82 (147.1–266.5)	142.6 (99.8–203.9)
⩽5	199	4441.0	44.81 (29.7–66.2)	37.6 (31.5–44.7)

a Published in: [16].
